# Soft fibrin matrix downregulates DAB2IP to promote Nanog-dependent growth of colon tumor-repopulating cells

**DOI:** 10.1038/s41419-019-1309-7

**Published:** 2019-02-15

**Authors:** Meng Zhang, Cong Xu, Hai-zhou Wang, Ya-nan Peng, Hai-ou Li, Yun-jiao Zhou, Shi Liu, Fan Wang, Lan Liu, Ying Chang, Qiu Zhao, Jing Liu

**Affiliations:** 1 Department of Gastroenterology, Zhongnan Hospital of Wuhan University, Wuhan, 430071 China; 2 Hubei Clinical Center & Key Lab of Intestinal & Colorectal Diseases, Wuhan, 430071 China; 3 Tongji hospital of Huazhong University of Science and Technology, Wuhan, 430030 China

## Abstract

Colon cancer stem cells (CSCs) have been shown to be responsible for the recurrence and metastasis of colorectal cancer (CRC). As a crucial microenvironmental factor, extracellular matrix (ECM) stiffness is known to affect the stemness of CSCs. Recently, fibrin deposition in the stroma of CRC was demonstrated to be responsible for tumor development. In this study, we used salmon fibrin gel to provide a 3D ECM for colon cancer cells and investigated its effects on cell growth as well as the underlying mechanisms. Compared with stiff 420 Pascal (Pa) and 1 050 Pa gels, 90 Pa soft fibrin gel was most efficient at isolating and enriching tumor colonies. The size and number of colony formation negatively correlated with gel stiffness. Specifically, these tumor colonies exhibited efficient tumorigenicity, upregulated stem cell markers, and had anti-chemotherapeutic properties and were thus named tumor-repopulating cells (TRCs). More importantly, the self-renewal molecule Nanog was sharply induced in 3D-cultured colon TRCs; further, Nanog siRNA significantly inhibited colony formation, suggesting the indispensable role of Nanog in TRC growth. A subsequent mechanistic study illustrated that Nanog expression could be modulated through fibrin gel stiffness-induced DAB2IP/PI3K/FOXA1 signaling in colon TRCs.

## Introduction

Colorectal cancer (CRC) is one of the most common causes of cancer-related death worldwide, and its morbidity is increasing sharply in the young population^1^. Accumulating evidence has demonstrated the existence of colon cancer stem cells (CSCs) and their profiles are highly prognostic for CRC patients^2^. Colon CSCs are a small population of tumor cells that feature immature cell markers, self-renewal properties, chemotherapy resistance, and secondary tumor-formation ability^3^. CSCs can even arise from nonstem/differentiated or chemically treated cells^4,5^. Thus, the origin of colon CSCs and corresponding regulatory mechanisms are still not fully understood.

Normal intestinal stem cells (ISCs) have two distinct populations: quiescent +4 cells (BMI1^+^, HOPX^+^, TERT^+^, and LRIG1^+^) and proliferative Lgr5^+^ cells^6^. Notably, researchers found bidirectional interconversion of the two ISC subtypes, as well as the transition of Lgr5^+^ cells into Lgr5^−^ and +4 cells upon certain stimulations^7^. Human CD133^+^ CRC cells were originally identified as resembling malignant tumors in mice^8,9^. Since then, a number of surface markers, including CD44v6^+^, Lgr5^+^, and EphB2^high^, were also discovered in colon CSCs^10–12^. Due to the heterogeneity of CSCs, biomarkers are sometimes controversial and unreliable for evaluation of colon CSCs. For example, they could be dynamically modulated by microenvironmental niches, such as tumor-associated cells, soluble cytokines/chemokine^5^, microbiota^13^, and noncellular supportive matrix (tumor stroma)^14^. Under microenvironmental stimulations, final interconversion between cancer progenitors and stem cells could be provoked through genetic and epigenetic regulation. For example, chemotherapy was reported to promote Lgr5^+^ and Lgr5^−^ CSC interconversion^15^. Additionally, tumor-associated fibroblasts could either reprogram CSCs or promote their self-renewal via secreting HGF^3^ or IL-17A^16^, respectively.

The noncellular supportive matrix is composed of proteoglycans, hyaluronic acid, and fibrous components, which could independently contribute to tumor differentiation and function^17^. For mouse embryonic stem cells, the soft extracellular matrix (ECM) was required to maintain their self-renewal and pluripotency, while hard matrix promoted cell differentiation^18^. Similarly, mesenchymal stem cells could differentiate into a neurogenic lineage with soft substrate, but become myogenic and osteogenic lineages within hard substrate^19^. In addition to nonmalignant cells, ECM stiffness also affects the fate of malignant cells^20^. In a previous study, we used fibrin gel to conduct 3D culture of tumor cells, the elastic stiffness of which was calculated by Pascal (Pa). We demonstrated that 90 Pa (1 mg/ml) soft fibrin gel could promote the growth and selection of multicellular colonies of melanoma^21^. These melanoma colonies had similar features as CSCs and were functionally termed tumor-repopulating cells (TRCs)^21^. Additionally, other tumor types also formed round colonies in 90 Pa fibrin matrix, such as hepatocarcinoma, ovarian cancer, and lymphoma^21^. Whether fibrin gel could be applied to enrich colon TRCs remains unknown.

Recently, fibrin(ogen) deposition was found to be increased within the stroma of the majority of tumor types. It promoted angiogenesis by supporting the binding of growth factors and facilitated tumor growth via thrombin/thrombin receptor interaction^22,23^. Additionally, it affected the stiffness of ECM and provided mechanical force to direct cell differentiation and function^24^. In the present study, different stiffness of fibrin matrix was applied to enrich colon TRCs. The CSC features of fibrin gel-cultured colon cancer cells were examined, such as colony formation, tumorigenicity, and chemo-resistance. Then, stem cell markers, differentiation markers, and self-renewal molecules were also analyzed. Furthermore, the regulatory mechanisms of colon TRCs were investigated.

DOC-2/DAB2 interactive protein (DAB2IP) is a member of the RAS–GTPase activating protein family. It is a tumor inhibitor in many cancers, such as colorectal, prostate, and bladder cancer^25^. Loss of DAB2IP was reported to promote CRC progression through NF-κB mediated Nanog and SOX2 upregulation^26^. Meanwhile, decreased levels of DAB2IP were also responsible for enhanced CSC phenotypes in prostate cancer through the PI3K/AKT/mTOR pathway^27^.

In this study, we use fibrin matrix to enrich colon TRCs and explore the effects of matrix stiffness on DAB2IP expression as well as downstream signaling transduction in colon TRCs. Our findings may provide a powerful tool for colon TRC study and highlight the physical-to-chemical signal transition in the modulation of cell fate and function.

## Results

### Soft fibrin matrix promoted the generation of multicellular colon cancer spheroids

**Fig. 1:**
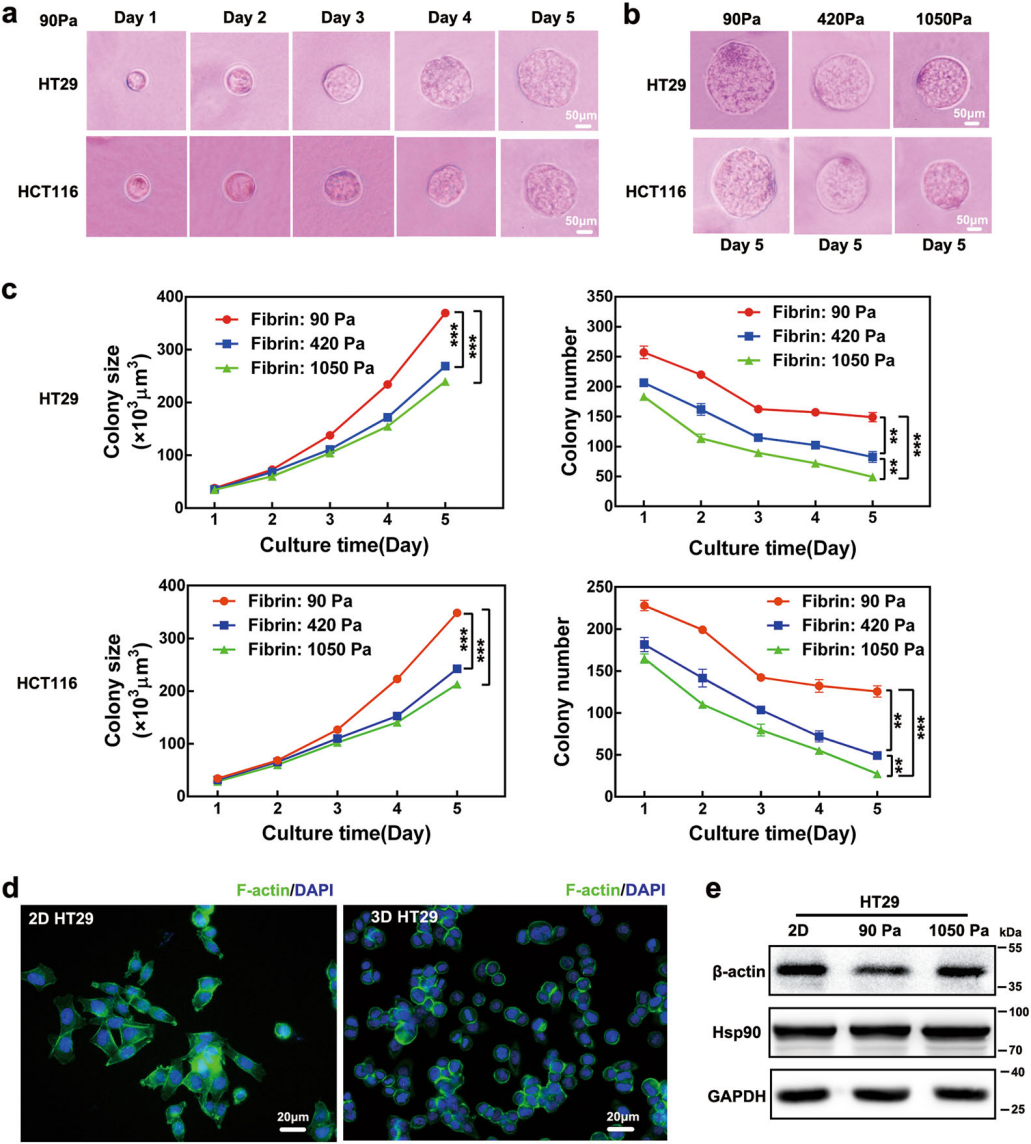
Colony formation of colon cancer cells in 3D soft fibrin gel. **a** Formation of multicellular colonies by single colon cancer cells. HT29 and HCT116 were cultured in 90 Pa soft fibrin gels from day 1 to day 5. **b**, **c** Colony formation of HT29 and HCT116 was reduced with increased fibrin gel stiffness. Means ± s.e.m., *n* = 6 (for 90 Pa gels) or 3 (for 420 or 1 050 Pa gels). **P < 0.01, ***P < 0.001. **d**, **e** Cytoskeleton detection in 2D and 3D HT29 cells. F-actin and β-actin were decreased in 3D HT29 cells compared with 2D controls. **d** Phalloidin staining of F-actin (green). Nuclei were stained with DAPI (blue). **e** β-Actin expression was analyzed by western blotting. GAPDH and Hsp90 were used as loading controls. Data shown in **d** and **e** represented three independent experiments. 2D rigid dish culture, 3D fibrin gel culture

As a scaffold protein of the ECM, fibrin deposition was recently shown to be enhanced within the stroma of CRC^22^. First, we tested fibrin expression in clinical CRC specimens and showed dramatically higher fibrin deposition in tumor sites compared with nontumor tissues (Figure S1). To further explore the effects of fibrin on CRC growth, a salmon fish fibrinogen and thrombin mixture was used to prepare a 3D fibrin gel^21^. Highly tumorigenic HT29 and HCT116 cells are the most commonly used in colon CSC research^28^. Therefore, we cultured HT29 and HCT116 cells in different stiffness of fibrin gels for 5 days, from soft 90 Pa to stiff 420 Pa and 1 050 Pa. In 90 Pa fibrin gel, cells were scattered individually, and 100–150 cells out of 1 250 cells survived and grew into round colonies from day 1 to day 5 (Fig. 1a). When gel stiffness increased to 420 and 1 050 Pa, both the size and number of tumor spheroids decreased dramatically over the course of the culture (Fig. 1b, c). To visualize the morphology and cytoskeleton of tumor spheroids, we examined the F-actin distribution of individual cells^29^. Phalloidin staining showed that 2D HT29 cells were irregular in morphology, with branched F-actin and multiple-stretched cell edges. However, 3D HT29 cells were in round shape, with less F-actin expression outlining the cell surface (Fig. 1d). In addition, cytoskeleton protein β-actin was also dramatically decreased in 3D HT29 compared with 2D HT29. More interestingly, the expression of β-actin was largely restored when the fibrin gel stiffness increased from 90 Pa to 1 050 Pa (Fig. 1e). These data suggested that 90 Pa soft fibrin gel was superior for colon cancer spheroid formation and caused the rearrangement and reduction of the cytoskeleton in colon cancer cells.

### Soft fibrin matrix enabled potent tumorigenicity of colon cancer spheroids

**Fig. 2:**
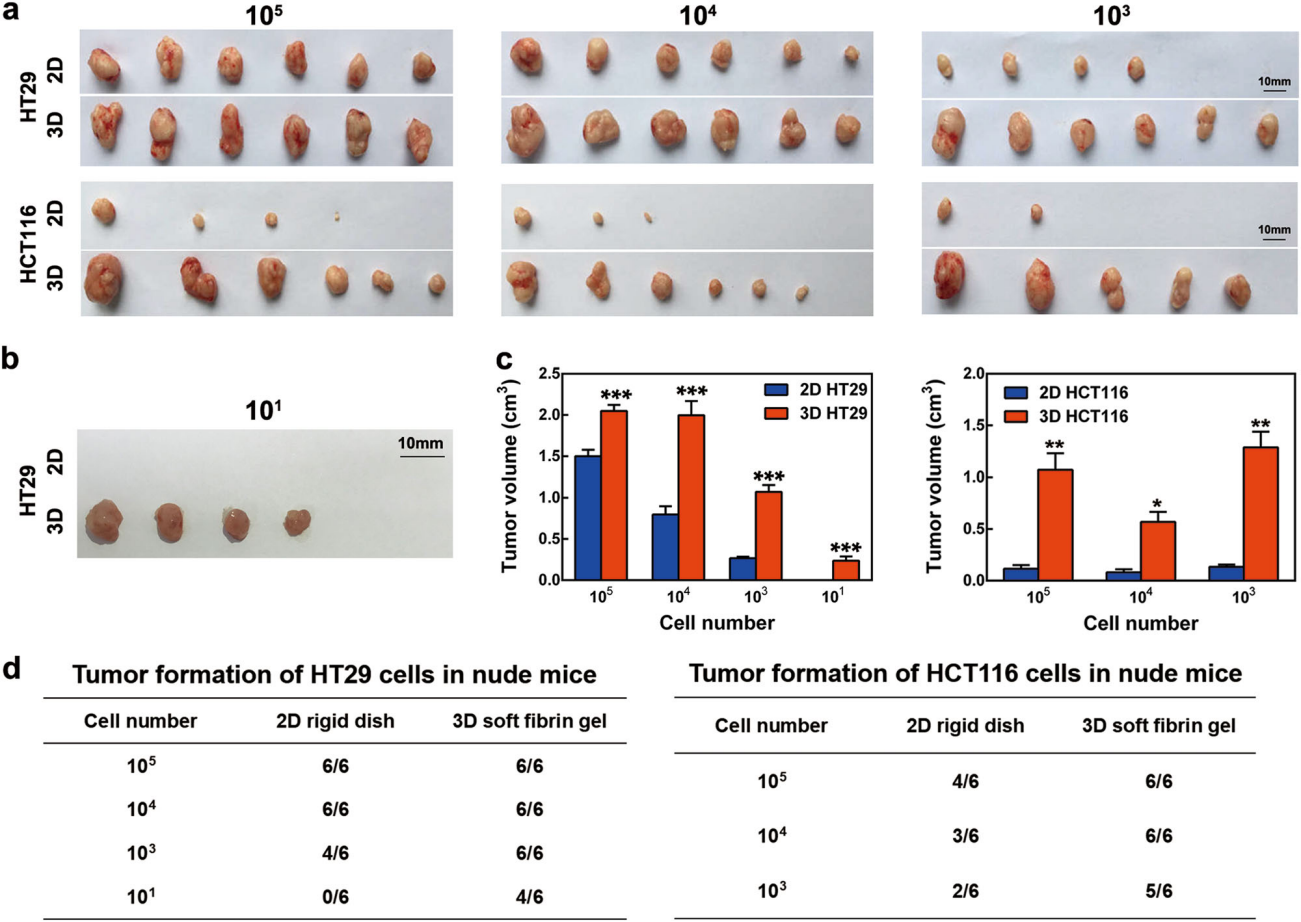
Tumorigenicity of colon cancer spheroids. **a** Formation of xenografts by 2D and 3D colon cancer cells. A total of 1 × 10^5^, 1 × 10^4^, or 1 × 10^3^ cells were subcutaneously injected in each BALB/c nude mouse. All mice were sacrificed 4 weeks after injection, and subcutaneous tumors were surgically excised. **b** Ten 2D or 3D HT29 cells were injected on the right flanks of 6-week-old BALB/c nude mice, respectively. **c** Tumor volume of 2D and 3D xenografts was calculated. Data are presented as the means ± s.e.m. (*n* = 6). *P < 0.05, **P < 0.01, ***P < 0.001, compared with 2D xenografts. **d** Tumor formation rate of 2D and 3D colon cancer cells. 2D rigid dish culture, 3D fibrin gel culture

We next determined whether 3D colon cancer spheroids were more efficient in tumorigenicity than 2D-cultured cells. Different numbers of 3D and 2D colon cancer cells were injected subcutaneously into BALB/c nude mice. After injection of 100,000 and 10,000 HT29 cells, the tumor formation rate was 100% (6/6) for both 3D and 2D cells (Fig. 2a and d). However, the volume of xenografts for 3D HT29 was significantly larger than that for 2D HT29 (Fig. 2c). At 1000 cells, the tumor formation rate was 100% (6/6) for 3D HT29, and 67% (4/6) for 2D HT29 (Fig. 2a and d). Notably, only 10 3D HT29 cells resulted in 67% (4/6) tumor formation, while no tumor formation was observed in 2D controls (Fig. 2b and d). These results indicated that colon cancer spheroids were more tumorigenic than their conventional counterparts. In parallel, we compared tumor formation between 3D and 2D HCT116 cells. After injecting 100,000, 10,000, and 1000 3D HCT116 cells, tumor formation rates were 100% (6/6), 100% (6/6), and 83% (5/6), respectively. The corresponding rates for 2D HCT116 were 67% (4/6), 50% (3/6), and 33% (2/6), respectively (Fig. 2a and d). Based on the high tumorigenic property, we functionally defined 3D HT29 and 3D HCT116 cells as colon TRCs. Together, our data indicated that 90 Pa soft fibrin matrix could promote potent tumorigenicity of colon TRCs.

### Soft fibrin matrix promoted Nanog-dependent growth and anti-apoptosis properties of colon TRCs

**Fig. 3:**
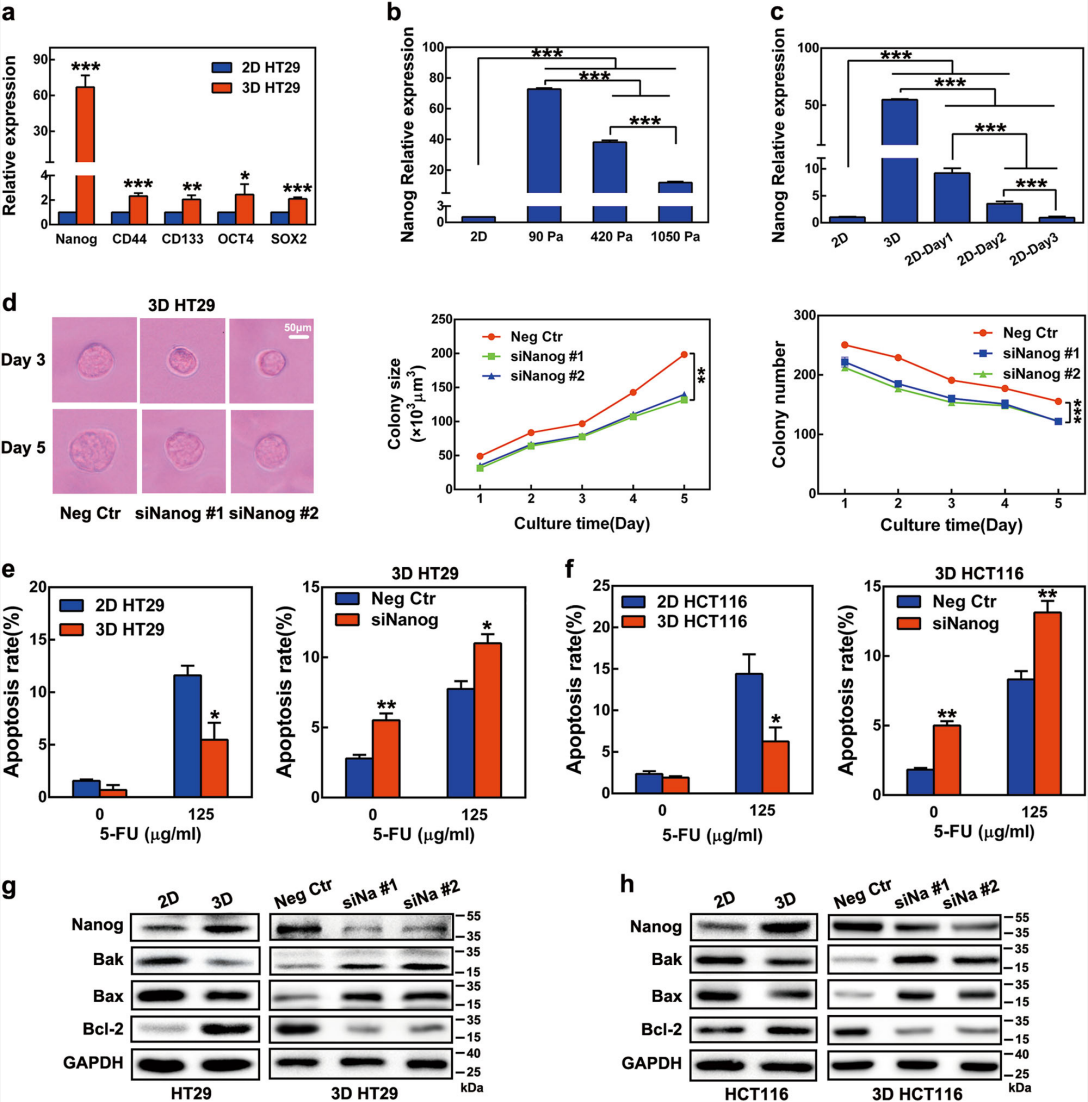
Impact of matrix stiffness on Nanog expression and the role of Nanog in stemness control of colon TRCs. **a** Expression of stem cell markers in 3D HT29 cells. mRNA expression of 2D and 3D HT29 cells was determined by real-time PCR. **b**, **c** Nanog expression in HT29 cells negatively correlated with matrix stiffness. Nanog mRNA was analyzed by real-time PCR. **b** Nanog expression in 2D rigid dish and 3D fibrin gels with different stiffness. **c** Effect of matrix switch on expression of Nanog mRNA in HT29 cells. **d** Nanog knockdown hindered colony formation of 3D HT29 cells. **e**, **f** Nanog protected colon TRCs from chemotherapeutic apoptosis. The 2D and 3D HT29 and HCT116 cells were treated with 125 μg/ml 5-FU for 18 h before FCM analysis. Nanog siRNA-transfected 2D HT29 and 2D HCT116 cells were cultured in fibrin gels. A total of 125 μg/ml 5-FU was added during the last 18 h of the 5-day culture. Then, the cells were tested for apoptosis by FCM. **g**, **h** Nanog directed modulation of apoptotic proteins in colon TRCs. Protein expressions were analyzed by western blotting. Nanog siRNA transfection and 3D cell culture were described as above. Data shown above are presented as the means ± s.e.m., *n* = 3; *P < 0.05, **P < 0.01, ***P < 0.001. 2D rigid dish culture, 3D fibrin gel culture, siNa #1 Nanog siRNA 1, siNa #2 Nanog siRNA 2

Next, the stemness of colon TRCs was analyzed. As shown by real-time PCR, stem cell markers, such as Nanog, CD133, CD44, OCT4, and SOX2, were upregulated in 3D HT29 and 3D HCT116 cells (Fig. 3a and Figure S2a). Other colon CSC markers, including Lgr5, DCLK1, and EphB2, were also upregulated in 3D HT29 cells (Figure S2b). In contrast, the differentiation markers CK20, CDX2, and CK7 were downregulated in 3D HT29 cells (Figure S2c). Since Nanog expression was over 40 times higher in both 3D HT29 and 3D HCT116 cells than in their 2D counterpart controls (Fig. 3a and Figure S2a), we asked what significant role it may play and how it was regulated. Nanog expression in HT29 cells was examined under different fibrin gel stiffness. Nanog was significantly enhanced at 90 Pa stiffness, decreased at 420 Pa, and was still lower at 1 050 Pa (Fig. 3b and Figure S3). Similarly, when 3D HT29 cells were seeded back to 2D rigid dishes, declination of Nanog was observed from day 1 to day 3 (Fig. 3c). Since Nanog was reported to promote the self-renewal of stem-like cancer cells^30^, we investigated the impact of Nanog knockdown on colony formation of colon TRCs. Nanog siRNAs markedly reduced colony size and the number of 3D HT29 cells (Fig. 3d). These data demonstrated that fibrin gel stiffness negatively regulated Nanog, thereby controlling colon TRC growth.

We further found that 3D HT29 and 3D HCT116 cells were resistant to 5-fluorouracil (5-FU)-induced apoptosis, exhibiting ~50% less than control cells (Fig. 3e, f). Such an effect could be largely blocked by knockdown of Nanog with siRNA (Fig. 3e, f). To explore the underlying mechanism, the expression of apoptosis factors was analyzed. We showed that pro-apoptosis Bak and Bax were inhibited, while anti-apoptosis Bcl-2 was enhanced in 3D HT29 and 3D HCT116 cells (Fig. 3g, h). However, silencing Nanog resulted in the recovery of Bak and Bax as well as the inhibition of Bcl-2 in these colon TRCs (Fig. 3g, h). The above findings revealed that Nanog protected colon TRC from chemotherapeutic apoptosis through apoptotic protein regulation.

### Soft fibrin matrix-mediated DAB2IP suppression was inversely correlated with Nanog expression in colon TRCs

**Fig. 4:**
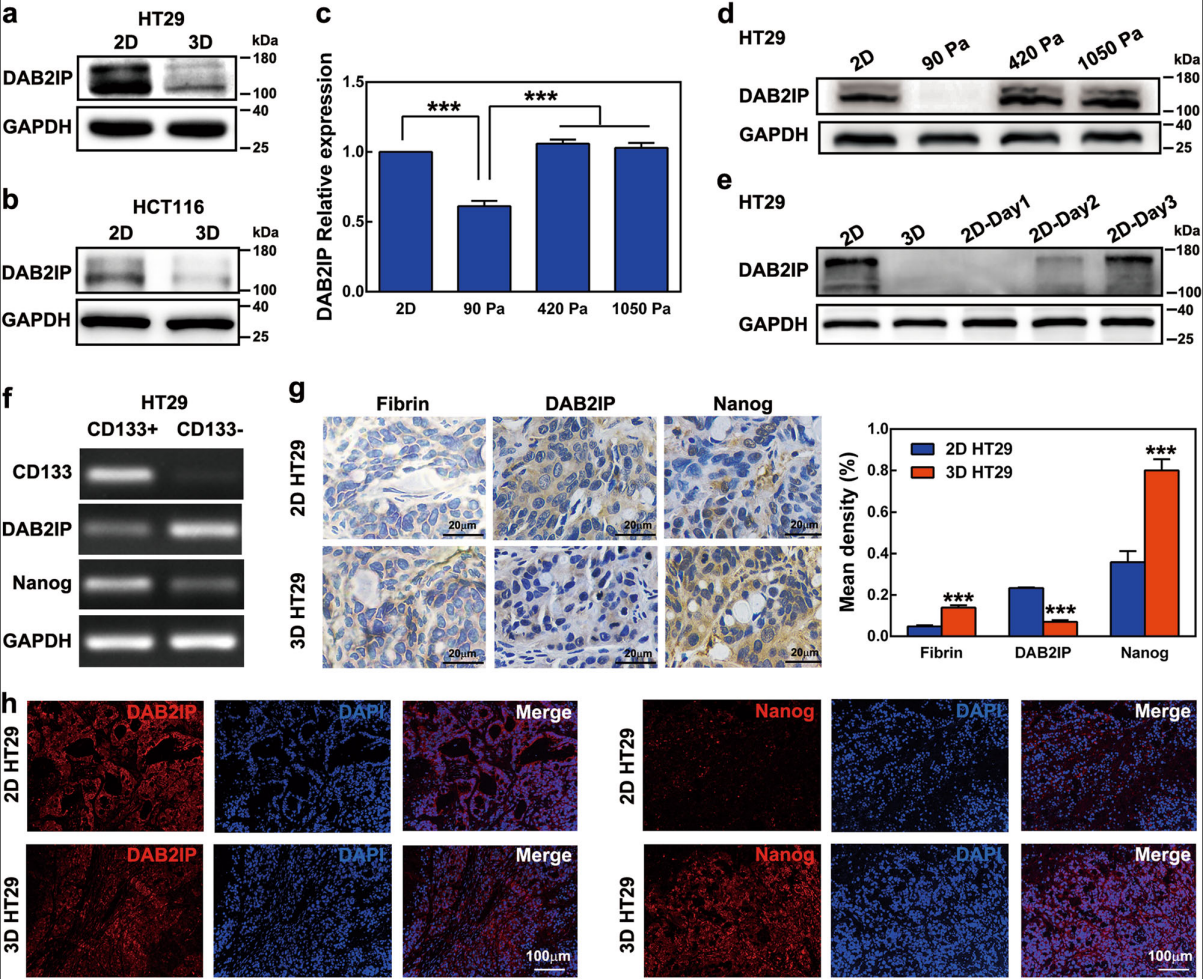
Impact of matrix stiffness on DAB2IP expression and the expression of DAB2IP and Nanog in colon cancer cells. **a**, **b** DAB2IP expression in 2D- and 3D-cultured colon cancer cells. Western blotting was used for protein detection. **c**, **d** Real-time PCR and western blotting analysis of DAB2IP in different stiffness of fibrin gel. **e** Effect of matrix switch on DAB2IP expression in HT29 cells. Western blotting was used for DAB2IP detection. **f** Expression of DAB2IP and Nanog in CD133^+^ and CD133^−^ HT29 cells, respectively. mRNA expression was analyzed by RT-PCR. **g** IHC staining of fibrin, DAB2IP, and Nanog. **h** IF staining of DAB2IP and Nanog in ex-vivo xenografts of 2D and 3D HT29 cells, respectively. Data shown above are presented as the means ± s.e.m., *n* = 3; ***P < 0.001

We further studied the mechanisms regulating Nanog expression. As a tumor suppressor gene, DAB2IP absence was shown to promote CSC-like signatures in CRC^26^. Similarly, DAB2IP was dramatically decreased in 3D HT29 and 3D HCT116 cells compared with 2D controls (Figure S4a and Fig. 4a, b). Such depression could be recovered when matrix stiffness was increased to 420 Pa and 1 050 Pa, or when cells were plated on plastic dishes (Fig. 4 c–e). The transcription factor Snail was reported to repress DAB2IP at the transcriptional level in HCT116 cells^31^. However, decreased Snail in 3D HT29 and 3D HCT116 could not explain the suppression of DAB2IP (Figure S4b), suggesting that complicated mechanisms in matrix stiffness mediated DAB2IP regulation.

Because CD133 is commonly used for colon CSC identification, we tested DAB2IP and Nanog expression in CD133^+^ HT29 cells. Compared with CD133^−^ counterparts, RT-PCR assays showed CD133^+^ HT29 cells had reduced DAB2IP and increased Nanog (Fig. 4f). This result coincided with those of 3D HT29 and 3D HCT116. We conducted immunohistochemistry (IHC) and immunofluorescence (IF) staining in subcutaneous xenografts of HT29 cells to evaluate the expression of Fibrin, DAB2IP, and Nanog. IHC results showed that fibrin was significantly increased in 3D HT29 tumors compared with 2D tumors (Fig. 4g). Additionally, IHC and IF showed that DAB2IP decreased dramatically in 3D HT29 tumors, but the opposite results were observed for Nanog (Fig. 4g, h). We thus proposed that DAB2IP participated in the negative regulation of Nanog.

### DAB2IP overexpression blocked colon TRC growth as well as Nanog-related anti-apoptosis regulation

**Fig. 5:**
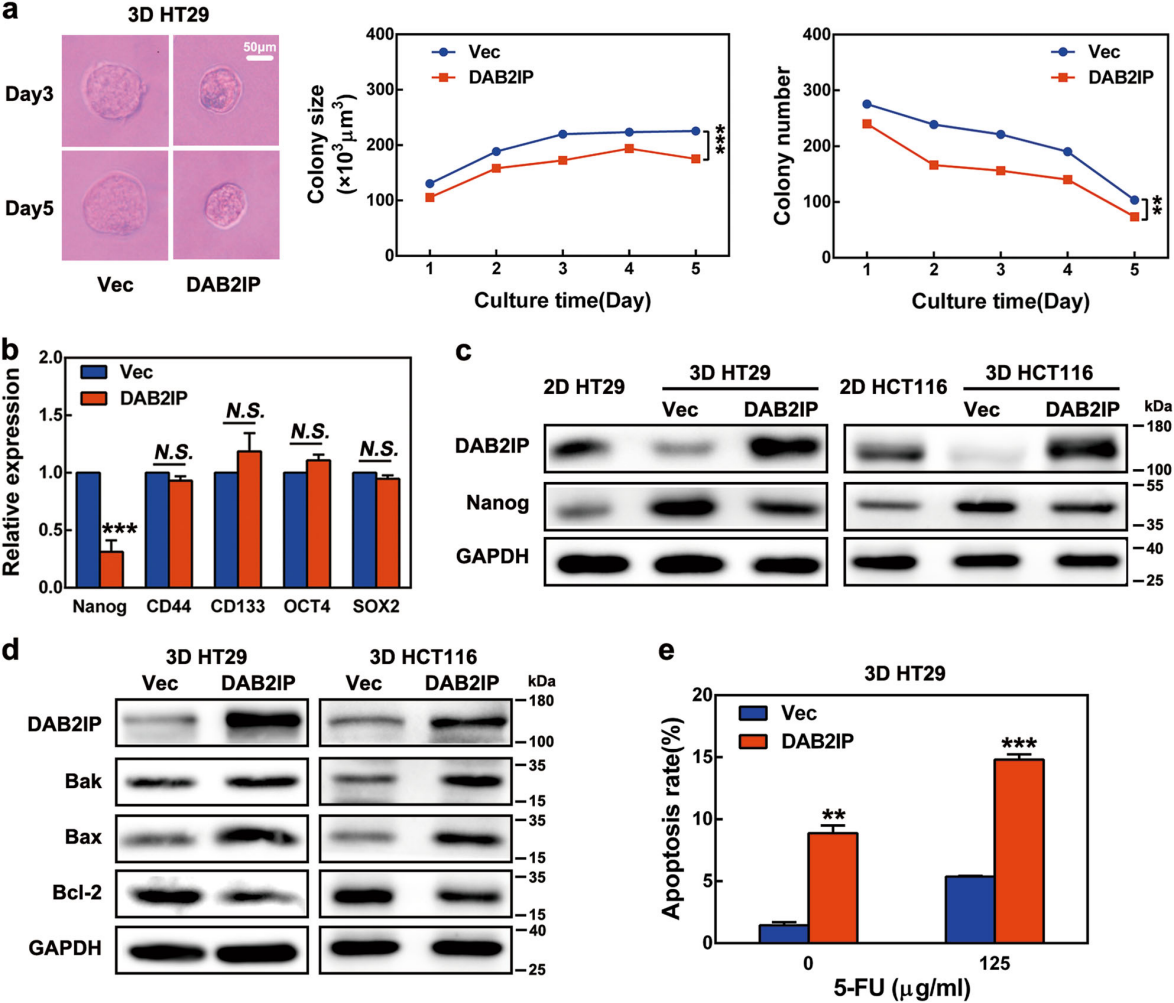
Effect of DAB2IP in colon TRC growth and Nanog regulation. HT29 and HCT116 cells were transfected with pcDNA3.1(+)-DAB2IP plasmids and cultured in fibrin gels. The pcDNA3.1(+) plasmids were used as control. **a** Impact of DAB2IP overexpression on colony formation of 3D HT29. **b** Effect of DAB2IP transfection on stem cell markers in 3D HT29 cells. Real-time PCR was used for mRNA examination. **c** DAB2IP overexpression inhibited Nanog expression in colon TRCs. **d**, **e** Effect of DAB2IP overexpression on apoptosis of colon TRCs. **d** DAB2IP and apoptotic proteins were analyzed by western blotting. **e** Apoptosis rate of 3D HT29 cells. Histogram was generated from FCM data. pcDNA3.1(+)-DAB2IP transfection and 3D cell cultures were performed as above. A total of 125 μg/ml 5-FU was added during the last 18 h of the 5-day culture. Data are shown as the means ± s.e.m., *n* = 3; **P < 0.01, *** P < 0.001, N.S. no significant difference, DAB2IP cells transfected with pcDNA3.1(+)-DAB2IP plasmids, Vec cells transfected with pcDNA3.1(+) plasmids

We next explored the impact of DAB2IP expression on colony formation of colon TRCs. Transfection of DAB2IP overexpressing plasmids in 3D HT29 cells resulted in significant reduction of both colony size and number (Fig. 5a). IF staining and real-time PCR confirmed that DAB2IP was overexpressed in most 3D HT29 cells after transfection (Figure S5a, b). To our interest, DAB2IP overexpression only caused a dramatic reduction of Nanog, leaving CD44 and other stem cell markers unaffected (Fig. 5b). Moreover, transfection of DAB2IP caused repression of Nanog protein in 3D HT29 and 3D HCT116 cells (Fig. 5c). Taken together, these findings demonstrated that DAB2IP negatively regulated colony formation and Nanog expression in colon TRCs. To verify these findings, DAB2IP blockade with siRNA was carried out in 2D HT29, which resulted in upregulation of Nanog (Figure S5c). However, Nanog knockdown had no impact on DAB2IP (Figure S5d and Figure S7). These results confirmed the one-way regulation of DAB2IP on Nanog expression.

As Nanog was demonstrated to be responsible for apoptosis regulation in the above studies, a similar role for DAB2IP was also investigated. We showed that DAB2IP overexpressing plasmids promoted the expression of Bak and Bax, but decreased the expression of Bcl-2 (Fig. 5d). Meanwhile, flow cytometry (FCM) showed significantly increased apoptosis in 3D HT29 cells or 5-FU-treated 3D HT29 cells, as a result of DAB2IP overexpression (Fig. 5e). These data demonstrated that DAB2IP was a tumor suppresser to promote apoptosis of colon TRCs.

### Soft fibrin matrix repressed DAB2IP expression to achieve PI3K/AKT activation and FOXA1/Nanog regulation in colon TRCs

**Fig. 6:**
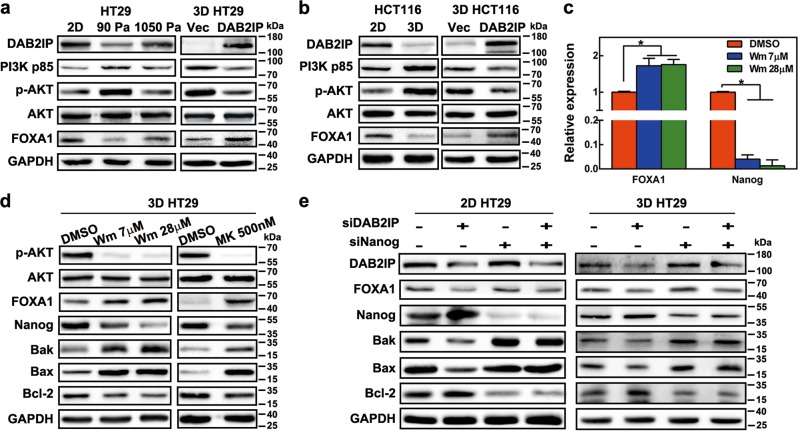
DAB2IP downstream signaling and its effect on Nanog-associated apoptosis regulation in colon TRCs. **a**, **b** Effect of matrix stiffness and DAB2IP expression on modulation of the PI3K/AKT pathway and FOXA1 in colon cancer cells. **c**, **d** Effect of PI3K and AKT inhibitors on expression of FOXA1 and Nanog in 3D HT29. Different concentrations of Wortmannin and MK2206 were added to the culture medium of 3D HT29 during the last 4 h or 24 h of culture, respectively. **c** Expression of FOXA1 and Nanog was analyzed by real-time PCR. Data are presented as the means ± s.e.m., *n* = 3; *P < 0.05. **d** Protein expression in 3D HT29 cells after Wortmannin and MK2206 treatment. **e** Effect of DAB2IP or/and Nanog knockdown on expression of FOXA1 and apoptotic proteins in 2D and 3D HT29 cells. Wm Wortmannin, MK MK2206

The regulatory mechanisms between DAB2IP and Nanog were further elucidated. In prostate cancer, loss of DAB2IP activates the PI3K/AKT pathway and elevates CSC phenotypes, while DAB2IP overexpression inhibits AKT phosphorylation (S473) by directly binding to PI3K-p85^32^. In this study, western blotting data showed that PI3K-p85 and p-AKT were overactivated in 90 Pa 3D HT29 and 3D HCT116 (Fig. 6a, b). This effect was blocked in stiff 1 050 Pa fibrin gel as well as in 2D rigid dishes (Fig. 6a). Transfection with DAB2IP overexpressing plasmids also inhibited the PI3K/AKT pathway in 3D HT29 and 3D HCT116 cells (Fig. 6a, b). Others have shown that p85β (one subtype of p85) and its encoding gene PIK3R2 were elevated in colon cancer and positively correlated with the activation of the PI3K pathway and tumor progression^33^. We found that PIK3R2 mRNA was also increased in 3D HT29 and could be reversed by transfection of DAB2IP plasmids (Figure S6a, b), suggesting that PI3K-p85 was transcriptionally activated. However, Nanog-specific siRNA did not impact either DAB2IP or PI3K/AKT expression (Figure S5c and Figure S7). These findings indicated that soft fibrin matrix-induced suppression of DAB2IP could promote PI3K/AKT activation as well as Nanog induction in 3D colon TRCs. In contrast to the activated PI3K/AKT pathway, the MAPK family number JNK was inactivated and could be recovered upon DAB2IP overexpression in 3D colon cancer cells (Figure S8a, b).

Transcription factor forkhead/winged-helix A1 (FOXA1) was reported to directly bind the Nanog promoter to repress its expression^34^ and play an important role as a regulator of cellular differentiation during embryonic development^35^. Compared with 2D rigid dishes, FOXA1 in HT29 and HCT116 cells was downregulated in 90 Pa soft fibrin gel and could be recovered in 1 050 Pa fibrin gel or by DAB2IP overexpressing plasmids (Fig. 6a, b and Figure S9a, b). We thus concluded that matrix stiffness could negatively regulate FOXA1 to promote Nanog expression via suppressing DAB2IP.

### DAB2IP negatively regulated Nanog expression via the PI3K/FOXA1 signaling pathway

To clarify the correlation among PI3K, FOXA1, and Nanog, we used PI3K and AKT inhibitors in 3D HT29 cells. Our data indicated that both the PI3K inhibitor Wortmannin^36^ and the AKT inhibitor MK2206^37^ increased FOXA1, repressed Nanog, and subsequently upregulated Bak and Bax as well as inhibited of Bcl-2 (Fig. 6c, d). Thus, these results demonstrated that DAB2IP could modulate FOXA1 and Nanog expression via the PI3K/AKT pathway.

**Fig. 7:**
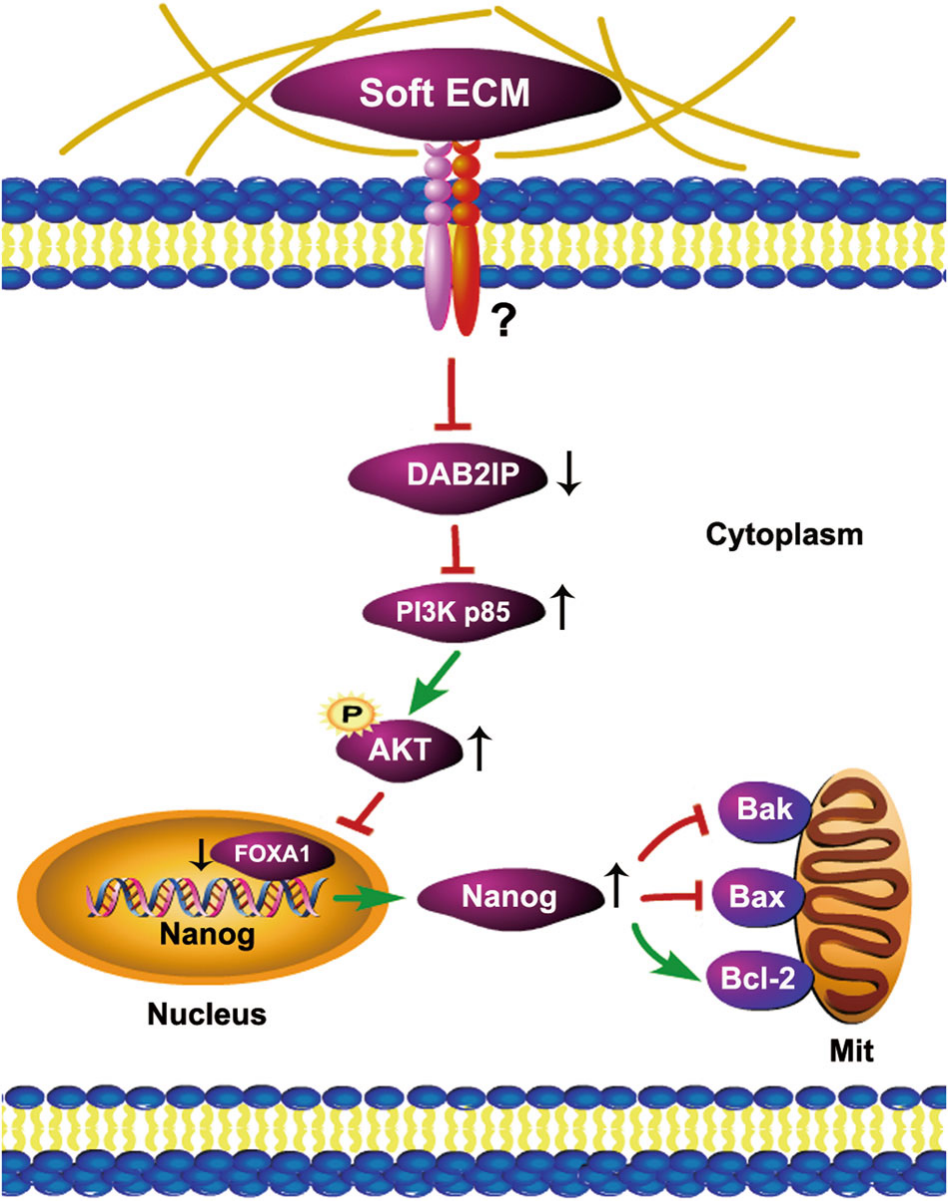
A schematic chart of fibrin matrix-mediated regulatory mechanisms in colon TRCs

Finally, the DAB2IP/PI3K/FOXA1 axis in Nanog regulation was further confirmed using DAB2IP siRNA and Nanog siRNA. We showed in both 2D and 3D HT29 cells that silencing DAB2IP resulted in FOXA1 inhibition and Nanog upregulation, followed by Bak/Bax inhibition and Bcl-2 activation (Fig. 6e and Figure S10a, b). In contrast, silencing Nanog only caused increased Bak/Bax and decreased Bcl-2, while not affecting DAB2IP or FOXA1 expression (Fig. 6e and Figure S10a, b). Moreover, silencing DAB2IP and Nanog together induced FOXA1 inhibition and apoptotic protein changes (Fig. 6e and Figure S10a, b). To conclude, our data revealed that 90 Pa soft fibrin matrix could suppress DAB2IP expression to facilitate PI3K/FOXA1/Nanog-mediated colon TRC enrichment (Fig. 7).

## Discussion

The incidence of CRC is increasing worldwide, and colon CSCs are responsible for the poor outcomes of patients^2^. At present, methods used for colon CSC expansion include multicellular spheroid culture^38^ and organoid culture^39^. These two methods cultivate single-cell suspension and intestinal crypt organoids (Matrigel) in serum-free medium supplemented with EGF/FGF-2 and EGF/R-spondin-1, respectively. In the present study, we used 3D fibrin gels to mimic the increased fibrin deposition in CRC^22^. We started with 90–1 050 Pa fibrin gels and demonstrated that 90 Pa was the optimal stiffness for spheroid formation of HT29 and HCT116 cells. Meanwhile, reduced F-actin and β-actin revealed reduced cytoskeleton assembly in 3D HT29 cells. Tumor cells with soft cytoskeletons were demonstrated to have enhanced deformability, aggravated tissue invasion, and improved metastasis^40,41^. The percentage of CD133^+^ CSCs in CRC patients was reported to be ~0.3–3%^38^. In our study, the formation rate of 3D colon TRCs in 90 Pa fibrin gel was 8–12%. This large difference indicated the reprogramming of colon cancer cells under the stimulus of soft matrix. The exact mechanisms require further elucidation.

We next showed that 3D HT29 and 3D HCT116 cells were more efficient in tumorigenicity than 2D control cells, and were therefore termed colon TRCs. Unlike HT29 cells, the tumor volume varied greatly within each single group of HCT116 cells, regardless of 3D or 2D culture. Compared with 2D HCT116, most 3D HCT116 generated larger tumors. However, the size discrepancy between the largest and smallest tumor in the same group of 3D HCT116 indicated great heterogeneity within individual cells. Of note, 10,000 3D HCT116 cells formed smaller tumor volumes than 1000 cells. Other factors, such as the handling of tumor inoculation and heterogeneity of mouse models may also responsible for the above variations.

Stem cell markers were upregulated while differentiation markers were downregulated in 3D HT29 cells. Specifically, Nanog was markedly increased compared with other stem cell makers. As a transcription factor, Nanog was originally identified as a key regulator of embryonic development and cellular reprogramming^42^. We showed that Nanog could be largely induced by soft fibrin matrix. This result coincided with a recent finding that increased Nanog was achieved in 3D mesenchymal stem cells by hanging-drop culture^43^. On the other hand, Nanog siRNA significantly inhibited the colony number and size of 3D HT29. Furthermore, FCM and western blotting showed anti-apoptosis effects of Nanog through repressing Bak/Bax and inducing Bcl-2. A similar result in human gastric cancer was also observed in another report^44^. These data strongly suggested the predominant role of Nanog in colon TRC growth.

The tumor suppressor gene DAB2IP attracted our interest in the mechanistic study of Nanog regulation. Previously, DAB2IP had been investigated in CRC and other cancers. Loss of DAB2IP could induce epithelial–mesenchymal transition in CRC and prostate cancer^45^. However, conditional DAB2IP overexpression sensitized prostate cancer cells to chemotherapeutics^46^. In this study, DAB2IP expression was repressed in 3D HT29 and 3D HCT116 and was negatively correlated with matrix stiffness. Tumor xenografts of 3D HT29 also showed reduced DAB2IP compared with those of 2D HT29. Additionally, CD133^+^ HT29 cells also expressed downregulated DAB2IP and upregulated Nanog. However, restoration of DAB2IP induced a serious reduction in both the number and size of 3D H29 colonies. Moreover, DAB2IP overexpression in 3D HT29 and 3D HCT116 specifically repressed Nanog expression and promoted the recovery of Bak and Bax, as well as inhibition of Bcl-2. These findings indicated the inhibitory role of DAB2IP in Nanog-mediated protection of colon TRCs.

DAB2IP is involved in PI3K/AKT signaling in prostate cancer and breast cancer^32^. The stemness of breast CSCs was sustained through PI3K/AKT-mediated Nanog expression^47^. We showed that significant activation of PI3K/AKT in colon TRCs could be blocked by DAB2IP overexpression. Because FOXA1 was reported to suppress Nanog expression^34^, we then proved that DAB2IP could inhibit Nanog expression via the PI3K/FOXA1 signaling pathway.

However, some issues remain to be further elucidated. First, the exact mechanism of DAB2IP regulation in colon TRCs is still unclear. Upstream force transducers should be screened, such as integrins and FAK. Moreover, people showed that JNK could promote Nanog transcription in HTC116^48^, and inhibition of JNK suppressed Nanog expression in chemo-resistant K562 and KB cancer cells^49^. In our study, inactivated p-JNK and enhanced Nanog in 3D HT29 and 3D HCT116 cells indicated the complexity of ECM dominated physical modulation. The underlying reasons remain unknown. Finally, other molecules involved in DAB2IP and Nanog regulation are worth further investigation.

## Conclusions

This study demonstrated that soft fibrin gel could promote growth and self-renewal of colon TRCs through the DAB2IP/PI3K/FOXA1/Nanog signaling axis. Successful use of soft fibrin gel in CSC enrichment will accelerate the study of CSC properties, as well as the exploration of novel tumor therapies.

## Materials and methods

### Cell lines and 2D cell culture

Human colon cancer cell line HT29 and HCT116 were obtained from China Center for Type Culture Collection (CTCC, Wuhan, China). For conventional 2D culture, cells were seeded on rigid dish with DMEM (Invitrogen, USA) containing 10% fetal bovine serum (FBS) (Hyclone, USA) at 37 °C with 5% CO_2_. Cells were passaged every 3–4 days with 0.25% Trypsin (Hyclone, USA).

### 3D fibrin gel cell culture

For 3D cell culture, fibrin gels were fabricated as previously described^21^. In brief, salmon fibrinogen and thrombin were purchased from Searun Holdings (San Diego, CA, USA). Fibrinogen and cell solution were 1:1 mixed to make 1, 4, or 8 mg/ml fibrinogen/cell solution, corresponding to 90, 420, and 1 050 Pa in elastic stiffness^21^. Next, 250 μl of fibrinogen/cell mixture and 5 μl of thrombin (100 U/ml) were well mixed to coat 24-well plates, which were then incubated for 30 min in a 37 °C CO_2_ incubator. Finally, DMEM with 10% FBS was added. After 5 days, tumor spheroids were harvested using Dispase Π (Roche, Switzerland). For each cell culture experiment, at least three independent experiments were performed.

### Gel stiffness calculation

Fibrin gels of 1, 4, and 8 mg/ml correspond to shear moduli of 30, 140, and 350 Pa, respectively, as measured by an RFS III fluid spectrometer rheometer^50^. The transformation between elastic stiffness (E) and shear modulus (G) is by the formula G = E/2(1 + μ), in which μ, a constant, represents Poisson’s ratio. Poisson’s ratio of fibrin equals 0.5, so 1, 4 and 8 mg/ml fibrinogen correspond to 90 Pa, 420 Pa, and 1 050 Pa, respectively.

### Colony number and size assay

A total of 1 250 HT29 or HCT116 cells were seeded in fibrin gel and cultured for 5 days. Colony number was counted per well under the white light of an inverted microscope (Olympus, Japan). Images were taken at day 5 by CCD camera under the white light of a fluorescence microscope (Olympus, Japan) (200 × ). The colony size was automatically analyzed by ImageJ software. At least three wells were counted at each condition every day.

### F-actin staining

F-actin expression and distribution were examined in 2D and 3D HT29 cells. The 2D HT29 cell suspension was prepared from a rigid dish culture and seeded on coverslips at a density of 4 × 10^4^ per well; 3D HT29 spheroids from 90 Pa fibrin gel were disseminated to make single-cell suspensions and then seeded on coverslips. After 8 h, 2D and 3D HT29 cells were fixed with 4% PFA, permeabilized and blocked with 0.1% Triton X-100 and 5% BSA. F-actin was detected with Alexa Fluor 488-phalloidin (1:400; Life Technologies, USA); nuclei were stained with DAPI. Images were acquired using a fluorescence microscope (Olympus, Japan).

### Real-time PCR

Total RNA of either 3D colon TRCs or 2D conventional cells was extracted using TRIzol reagent according to the supplier’s instructions (Invitrogen, Carlsbad, CA, USA). Reverse transcription (RT) was performed using Transcript First-strand cDNA Synthesis Super Mix (Roche, Switzerland). Real-time PCR was conducted with SYBR^®^ Premix Ex Taq™ II (Tli RNaseH Plus) (TaKaRa, Japan) on ABI QuantStudio™ 6 Flex System (USA). Data were analyzed with the comparative CT method for relative gene-expression quantification against GAPDH. The primer sequences are provided in Table S1.

### Western blotting

Cells were lysed with NP40 Lysis buffer (Beyotime, China) and protein concentration was tested by BCA kits (Thermo, USA). Protein lysates were separated by SDS-PAGE, then transferred to PVDF membranes (Millipore, USA) and blocked with 5% fat-free milk. The membranes were incubated overnight at 4 °C with the following primary antibodies: DAB2IP (Proteintech, USA), PI3K-p85 (CST, USA), p-AKT (CST, USA), AKT (CST, USA), p-JNK (CST, USA), JNK (CST, USA), Nanog (Proteintech, USA), Bak (CST, USA), Bax (CST, USA), Bcl-2 (Proteintech, USA), β-actin (Servicebio, China), FOXA1 (Abcam, UK), and GAPDH (Servicebio, China). Anti-rabbit IgG-HRP (Servicebio, China) secondary antibody was then applied. Chemiluminescent signals were detected by Enhanced Chemiluminescence (Thermo, USA). Results were confirmed by at least three independent experiments.

### Animal studies

The 6-week-old BALB/c nude mice were purchased from Beijing Vital River Laboratory Animal Technology Company (China). Six mice per group were randomly allocated, and all animal experiments were conducted in accordance with standard procedures and approved by the Animal Ethics Committee of Wuhan University. The 2D and 3D colon cancer cell suspensions were subcutaneously injected on the right flanks of mice. After 4 weeks, mice were killed. Images of xenografts were recorded by digital camera (Canon, Japan). Tumor volume was calculated by the formula (A*B^2^)/2, where A stood for the long dimension and B represented the short dimension.

### Apoptosis analysis by FCM

Colon cancer cells were collected and stained with Annexin-V FITC/PI. Apoptosis detection kits (BD Biosciences, USA) were used according to the manufacturer’s protocol. Flow cytometry (FCM) (BD Biosciences) was used to detect apoptotic cells, and the data was analyzed by FlowJo software (TreeStar, USA).

### Plasmids and siRNA transfection

Nanog siRNAs and DAB2IP siRNAs were purchased from Ribobio (Guangzhou, China). Corresponding siRNAs (50 nM) were transfected to 2D HT29 and 2D HCT116 cells. After 48 h, colon cancer cells were cultured in 90 Pa fibrin gel for 5 days. Scrambled siRNAs were used as control. siRNA sequences are listed in Table S2.

pcDNA3.1(+) and pcDNA3.1(+)-DAB2IP plasmids were gifted from Prof. Daxing Xie of Tongji Tongji Cancer Research Institute. Using Lipofectamine 2000 (Invitrogen, USA), pcDNA3.1(+)-DAB2IP, or pcDNA3.1(+) plasmids (2.5 μg per well) were transfected to 2D HT29 and 2D HCT116 cells following the manufacturer’s protocol, respectively. After 48 h, transfected colon cancer cells were cultured in 90 Pa fibrin gels for 5 days.

### CD133^+^ HT29 cell isolation

CD133^+^ HT29 cells were enriched by positive magnetic selection using the MACS cell separation system (Miltenyi Biotec, Germany). Single HT29 cells were labeled with magnetic CD133 microbeads (Miltenyi Biotec) at 4 °C for 30 min. Then, the cell suspension was loaded onto the prepared MS column in the magnetic field of MACS Separator. The magnetically labeled CD133^+^ cells were retained within the column. The unlabeled CD133^−^ HT29 cells run through. After removing the column from the magnetic field, the magnetically retained CD133^+^ HT29 cells was eluted.

### Immunohistochemistry and immunofluorescence staining

The subcutaneous xenografts were surgically excised from BALB/c nude mice, paraformaldehyde fixed and paraffin embedded, and sectioned. After dewaxing, rehydration, and antigen retrieval, the slides were blocked in 5% BSA for 40 min and incubated with primary antibodies at 4 °C overnight: Fibrin(ogen) (Novus, USA), DAB2IP (Proteintech, USA), Nanog (Proteintech, USA). Slides were then incubated with HRP-conjugated or CY3-conjugated anti-rabbit secondary antibody (Servicebio, China) for immunohistochemistry (IHC) or immunofluorescence (IF) assays. DAPI was used to stain the nucleus.

IHC images were acquired under white light of fluorescence microscope (Olympus, Japan) and Image-pro plus 6.0 software was used to quantify the corresponding staining. IF staining was recorded by fluorescence microscope (Olympus, Japan).

### Statistical analysis

The results were analyzed using GraphPad PRISM software (GraphPad Software Inc., USA). Two tailed Student’s *t* test was used to analyze statistical significance between groups: *P < 0.05, **P < 0.01, ***P < 0.001. *P* < *0*.05 was considered significant difference. All data were representative of multiple independent experiments.

## Supplementary information

Supplementary Information
