# Supplementary material for: Soft fibrin matrix downregulates DAB2IP to promote Nanog-dependent growth of colon tumor-repopulating cells
Source: Cell Death Dis. 2019 Feb 15;10(3):151. doi: 10.1038/s41419-019-1309-7 (PMC6377646; doi:10.1038/s41419-019-1309-7)
Supplement: Supplementary file 1 — Supplementary Information [file 41419_2019_1309_MOESM1_ESM.docx]

**Supplementary Information**

**Figures and Figure legends**

**
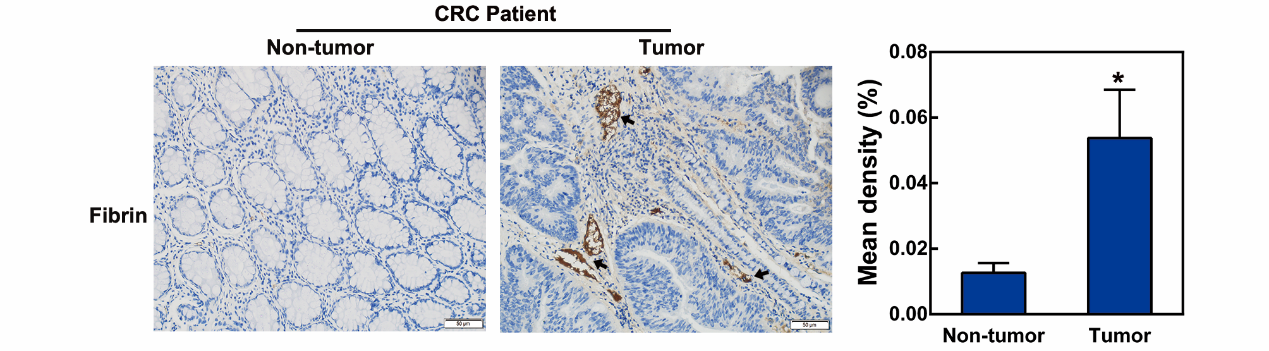
**

**Figure S1. Fibrin deposition in colon cancer specimen.** Fibrin expression was elevated in tumor site than in non-tumor tissue. Six cases of clinical colon cancer specimens and paired non-tumor tissues were collected from Zhongnan Hospital of Wuhan University and diagnosed by the Department of Pathology. Primary Fibrin(ogen) antibody (Novus, USA) and secondary HRP-conjugated anti-rabbit antibody (Servicebio, China) were used for IHC staining. Image-pro plus 6.0 software was used to quantify Fibrin staining. Data shown was presented as mean ± s.e.m., n = 6; **P<0.05*.

**
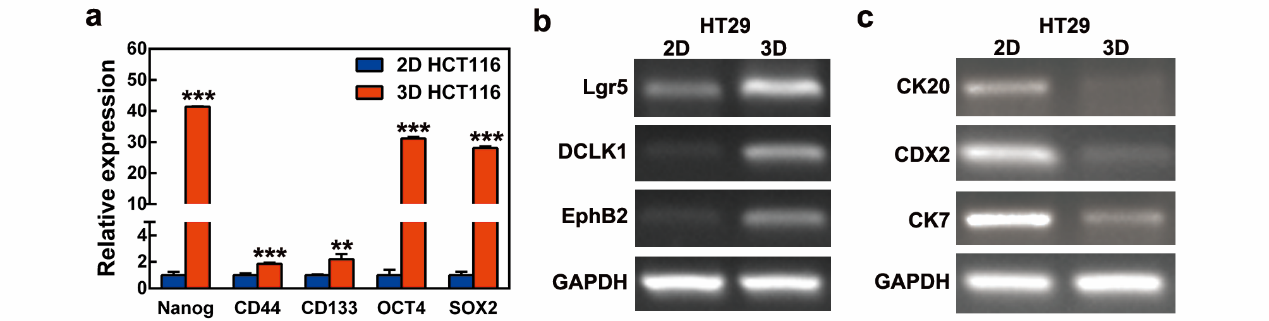
**

**Figure S2. The expression** **levels of stem cell markers and differentiation markers in colon TRCs.** **(a)** Total mRNA of stem cell markers in 3D HCT116 cells. 2D HCT116 cells were used as control. Data shown above was presented as mean ± s.e.m., n = 3; ***P<0.01, *** P<0.001.* **(b)** Stem cell markers and **(c)** differentiation markers in 2D and 3D HT29 cells were analyzed by RT-PCR, respectively. 2D: rigid dish; 3D: 90 Pa fibrin gel.


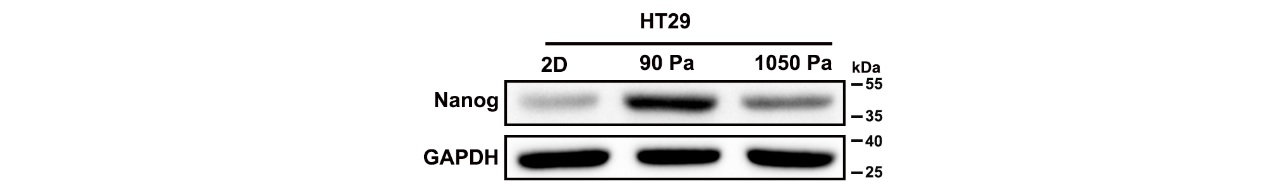


**Figure S3. Nanog expression in HT29 cells under different matrix stiffness.** Protein expression of Nanog was determined by Western blotting. HT29 cells were cultured in rigid dish and different stiffness of fibrin gels, respectively. Data was representative of three independent experiments. 2D: rigid dish; 90 Pa, 1 050 Pa: 90 Pa and 1 050 Pa fibrin gel.


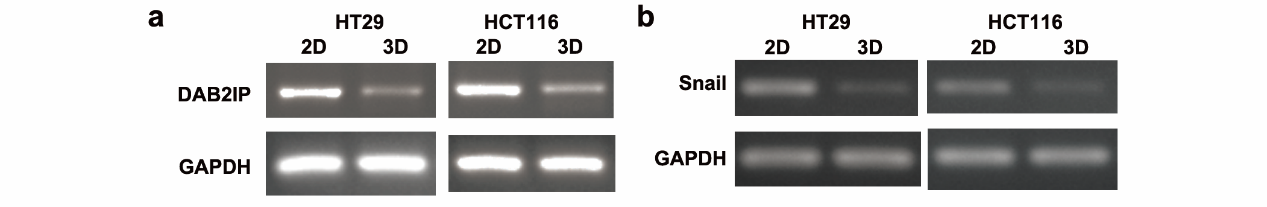


**Figure S4. mRNA expression levels of DAB2IP and Snail in colon TRCs.** RT-PCR was used to detect **(a)** DAB2IP and **(b)** Snail expression. Cells were maintained in rigid dish and 90 Pa fibrin gel, respectively. Data shown was representative of three independent experiments. 2D: rigid dish; 3D: 90 Pa fibrin gel.

**
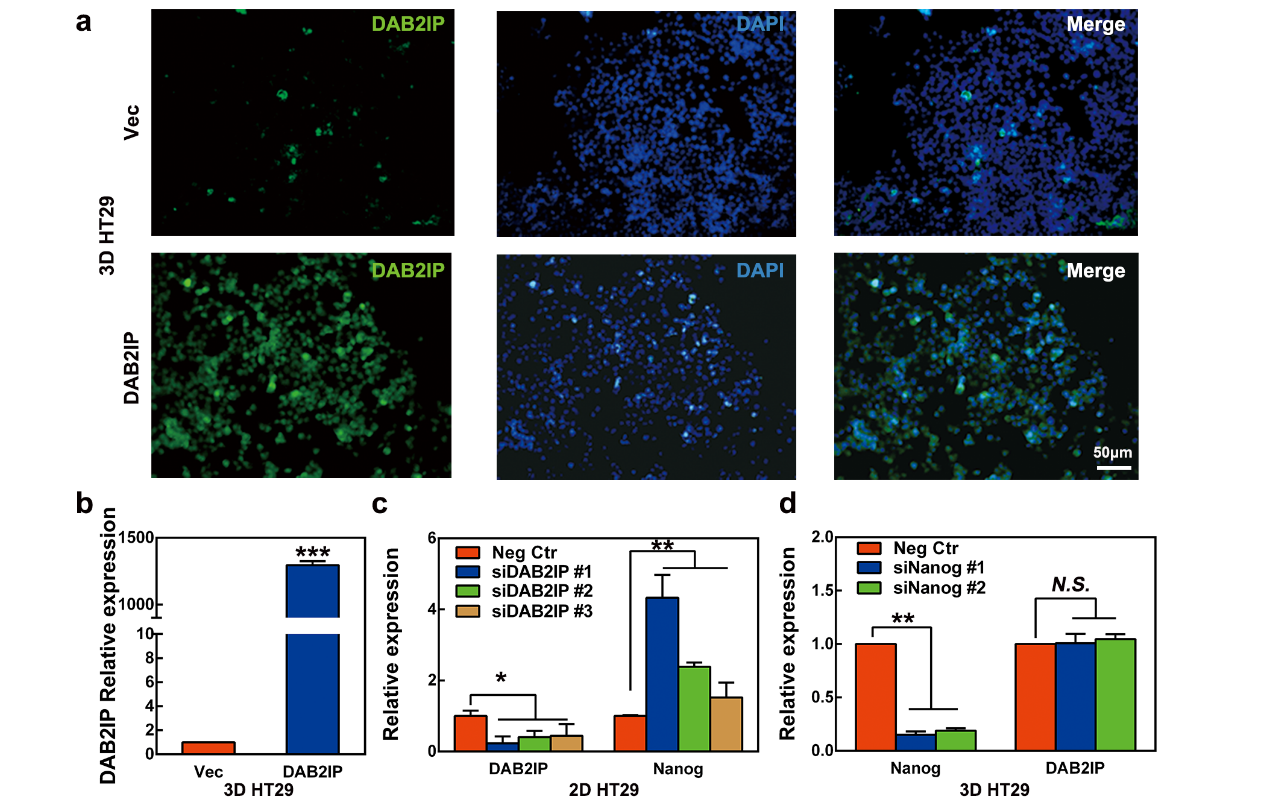
**

**F****igure S5. Effect of DAB2IP expression on Nanog expression in colon TRCs. (a)** Immunofluorescence staining of DAB2IP in 3D HT29 cells. pcDNA3.1(+)-DAB2IP plasmids (2.5 μg per well) were transfected to 2D HT29 cells. After 48 hours, cells were cultured in 90 Pa fibrin gels for five days. pcDNA3.1(+) plasmids were used as control. 3D HT29 spheroids from fibrin gel were disseminated to make single cell suspension, and then seeded on coverslips for Immunofluorescence staining. **(b)** Real-time PCR analysis of DAB2IP in 3D HT29 cells. **(c)** Real-time PCR of DAB2IP and Nanog in 2D HT29 cells transfected with DAB2IP siRNAs. **(d)** Expression levels of Nanog and DAB2IP in 3D HT29 cells with Nanog siRNA transfection. Real-time PCR was used to detect mRNA expression. Data shown above was presented as mean ± s.e.m., n = 3; ***P<0.01, ***P<0.001, N.S.: no significant difference.* Vec: pcDNA3.1(+) plasmid; DAB2IP: pcDNA3.1(+)-DAB2IP plasmid. 2D: rigid dish; 3D: 90 Pa fibrin gel.

**
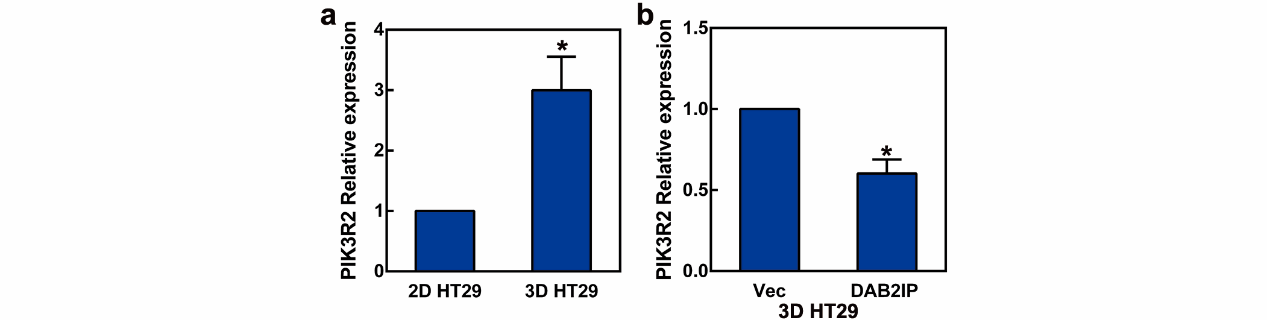
**

**Figure S6.** **PIK3R2 expression in HT29 cells**. **(a)** Real-time PCR was used for PIK3R2 mRNA examination. HT29 cells were cultured in rigid dish and 90 Pa fibrin gel, respectively. **(b)** Impact of DAB2IP overexpressing plasmids on PIK3R2 expression in 3D HT29 cells. 2D HT29 cells were transfected with pcDNA3.1(+)-DAB2IP plasmids and then cultured in 90 Pa fibrin gels. Data shown above was presented as mean ± s.e.m., n = 3; **P<0.05*. 2D: rigid dish; 3D: 90 Pa fibrin gel. Vec: pcDNA3.1(+) plasmid; DAB2IP: pcDNA3.1(+)-DAB2IP plasmid.


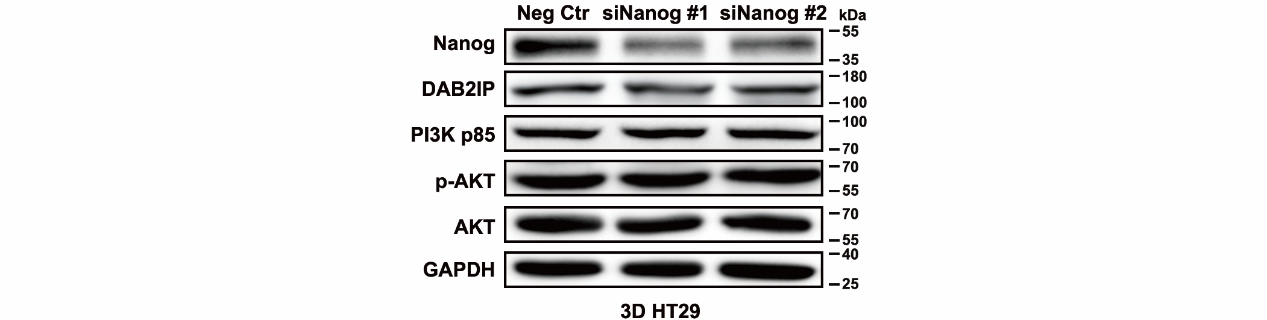


**Figure S7.** **Effect of** **Nanog knockdown on DAB2IP expression and PI3K/AKT activation in 3D HT29 cells.** Protein expression was analyzed by Western blotting. 2D HT29 cells were transfected with Nanog siRNAs and then cultured in 90 Pa fibrin gels. Scrambled siRNAs were used as control. Data shown was representative of three independent experiments.

**
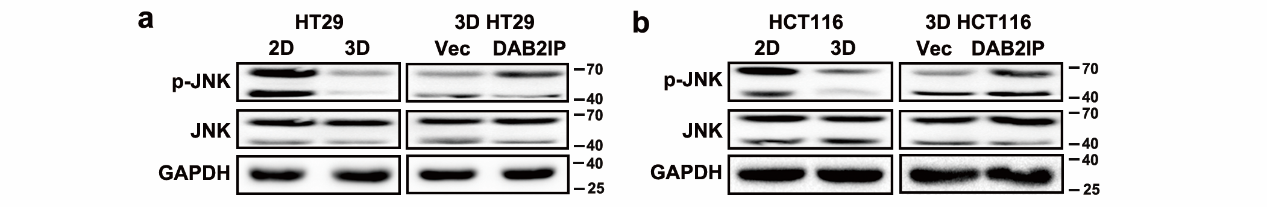
**

**Figure S8. Effect of matrix stiffness and DAB2IP expression on signal transduction in colon cancer cells.** Western blotting was used to detect protein expression of p-JNK. p-JNK expression and its regulation by DAB2IP in **(a)** HT29 and **(b)** HCT116 cells. Protein expression was analyzed by Western blotting.

**
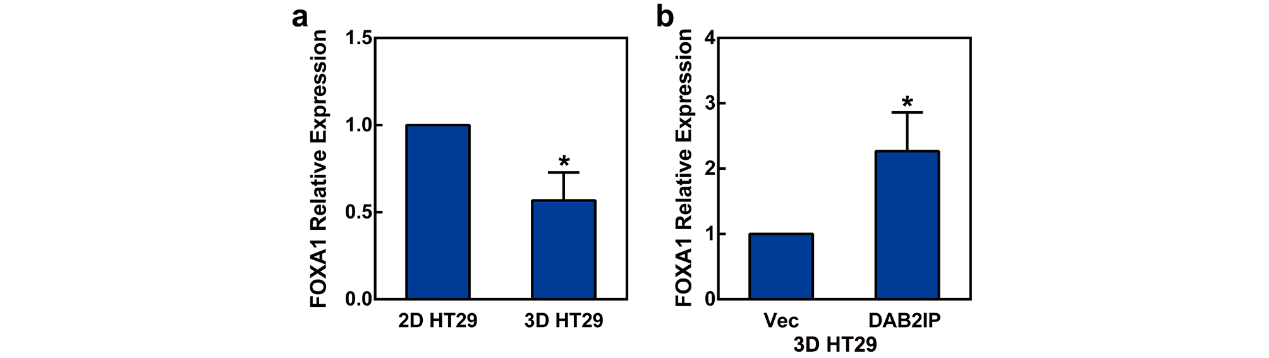
**

**F****i****g****u****re S9.** **F****O****XA1 expression in HT29 cells by real-time PCR assays.** **(a)** mRNA expression of FOXA1 in 2D and 3D HT29 cells. **(b)** Impact of DAB2IP over-expressing plasmids on FOXA1 expression in 3D HT29 cells. Data was presented as mean ± s.e.m., n = 3; **P<0.05*.


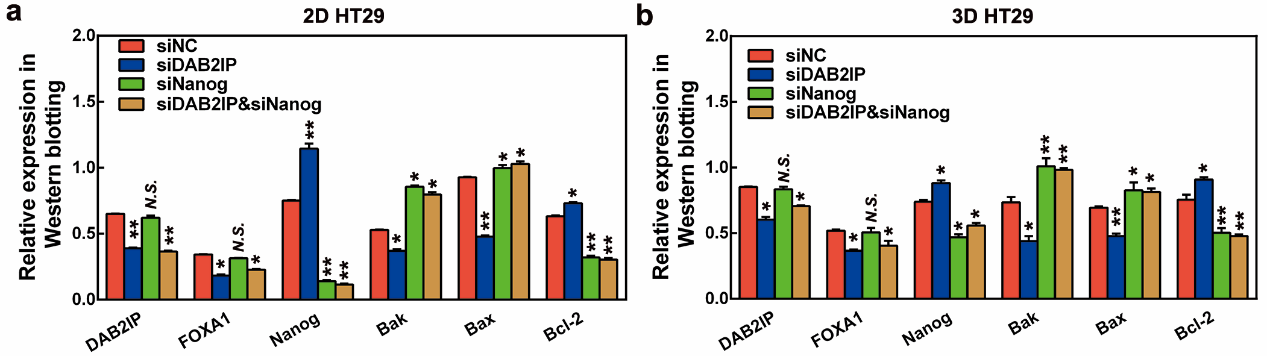


**Figure S10. Effect of DAB2IP or/and Nanog knockdown on FOXA1 and apoptosis protein expression in HT29 cells.** The gray intensity of protein expression in 2D and 3D HT29 was quantified by image J software. Data was presented as mean ± s.e.m., n = 3; **P<0.05, **P<0.01, N.S.: no significant difference.*

**T****a****b****le S1.** **Sequences of primers for Real-time PCR.**

| **Gene** |  | **Real-time PCR** |
| --- | --- | --- |
| **Hum-Nanog** | **5’Primer** | **CTCCAACATCCTGAACCTCAGC** |
|  | **3’Primer** | **CGTCACACCATTGCTATTCTTCG** |
| **Hum-CD44** | **5’Primer** | **CTGCCGCTTTGCAGGTGTA** |
|  | **3’Primer** | **CATTGTGGGCAAGGTGCTATT** |
| **Hum-CD133** | **5’Primer** | **AGTCGGAAACTGGCAGATAGC** |
|  | **3’Primer** | **GGTAGTGTTGTACTGGGCCAAT** |
| **Hum-OCT4** | **5’Primer** | **CCTGAAGCAGAAGAGGATCACC** |
|  | **3’Primer** | **AAAGCGGCAGATGGTCGTTTGG** |
| **Hum-SOX2** | **5’Primer** | **TACAGCATGTCCTACTCGCAG** |
|  | **3’Primer** | **GAGGAAGAGGTAACCACAGGG** |
| **Hum-DAB2IP** | **5’Primer** | **TCATCGCCAAGGTCACCCAGAA** |
|  | **3’Primer** | **CGCTGCATGTTGGTCCACTCAT** |
| **Hum-FOXA1** | **5’Primer** | **CCCTTTGTCCTCTCTACCCACA** |
|  | **3’Primer** | **GCTCCCTATAACTTATCTCTCCTCCA** |
| **Hum-Lgr5** | **5’Primer** | **CCCAGTTCCGTGCCATCACC** |
|  | **3’Primer** | **AACCGAGTTTCACCTCAGCTCTTCTTAT** |
| **Hum-DCLK1** | **5’Primer** | **GCACGGTTCTTTCTTCTTCA** |
|  | **3’Primer** | **GGGCGTCATCAGTACATCTT** |
| **Hum-EphB2** | **5’Primer** | **TCATCGCCGTGCGTGTCTTC** |
|  | **3’Primer** | **GCTTGATGGGTACATCCACCTCTT** |
| **Hum-CK20** | **5’Primer** | **AACTAACGGAGCTGAGACGC** |
|  | **3’Primer** | **CCTCCAGAGAGCTCAACAGC** |
| **Hum-CDX2** | **5’Primer** | **CTCGGCAGCCAAGTGAAAAC** |
|  | **3’Primer** | **TCAGAGAGCCCCAGCGT** |
| **Hum-CK7** | **5’Primer** | **AGGCGCAGTATGAGGAGATGGC** |
|  | **3’Primer** | **TGGCACGCTGGTTCTTGATGTT** |
| **Hum-Snail** | **5’Primer** | **GTGCCCTCAAGATGCACAT** |
|  | **3’Primer** | **CTTGACATCTGAGTGGGTCT** |
| **Hum-PIK3R2** | **5’Primer** | **TGCTTCAGGAACACTTGGAAG** |
|  | **3’Primer** | **AAGGTGCCATCGGGAGTGT** |

**T****a****b****le S2. Sequences of siRNA**

| **siRNA** |  | **Sequences** |
| --- | --- | --- |
| **hsa-Nanog** | **#1** | **GGAAGGCCTTAATGTAATA** |
|  | **#2** | **GCCGAAGAATAGCAATGGT** |
|  | **#3** | **GCATGCAGTTCCAGCCAAA** |
| **hsa-DAB2IP** | **#1** | **GGTGAAGGACTTCCTGACA** |
|  | **#2** | **GGGATAGGCTAAGGAGTAA** |
|  | **#3** | **GGAGCGCAACAGTTACCTG** |
